# A survey of antibiotic prophylaxis in adult cardiac surgery in the UK

**DOI:** 10.1186/1749-8090-10-S1-A215

**Published:** 2015-12-16

**Authors:** Aidil Syed, Tom Combellack, Prakash Nanjaiaj, Dheeraj Mehta, Subramamiam Balachandran

**Affiliations:** 1 Cardiothoracics, University Hospital of Wales, Cardiff, UK

## Background/Introduction

Antibiotic prophylaxis has been proven to reduce surgical site infection (SSI) rates in adult cardiac surgical patients. However, current guidelines are vague and in this era of increasing antibiotic resistance it is essential that we inform and develop a consensus opinion.

## Aims/Objectives

Our aim was to acquire an overview of antibiotic prophylaxis in adult cardiac surgical centres in the UK and compare this to national and international guidelines.

## Method

We developed a 10 point questionnaire and obtained results via telephone interview of on-call cardiac surgery registrars in all UK units in April 2015.

## Results

A total of 32/35 (91%) of on-call registrars responded, with the majority (21/32, 66%) being unaware of their local SSI rate. Only one cardiac centre used a surgeon-specific policy while the remaining 31 centres (97%) used a unit-specific policy. Although all units complied with guidelines, there was wide variation in practice. For indexed operations e.g. CABG and isolated valve procedures, the majority (21/32, 66%) of units used single antibiotic prophylaxis, most commonly cefuroxime (14/32, 44%). The remaining centres used combination antibiotic prophylaxis (11/32, 34%), most commonly flucloxacillin and gentamicin (8/32, 25%). Most units (23/32, 72%) prescribed prophylaxis for 24 hours post operatively, 3 units (9%) for 48 hours and 4 units (12.5%) administered only a single dose.

Approximately half of centres used MRSA skin preparation bundles even when MRSA status was unknown and 7 centres (22%) routinely used anti-MRSA antibiotic agents.

## Discussion/Conclusion

The results of this survey demonstrate that while all UK adult cardiac centres adhere to guidelines, there is significant divergence in policy between different centres.

While it is clear that large scale multi-centre studies are required to develop more specific guidelines, particularly in terms of antibiotic selection and duration, the results of this national survey will help to inform ongoing debate and guide future policy development.

